# Effects of Operational Parameters on the Low Contaminant Jarosite Precipitation Process-an Industrial Scale Study

**DOI:** 10.3390/ma13204662

**Published:** 2020-10-19

**Authors:** Ali Asimi, Khodakaram Gharibi, Emad Abkhoshk, Farhad Moosakazemi, Saeed Chehreh Chelgani

**Affiliations:** 1 Department of Mining and Metallurgical Engineering Yazd University, Yazd 89195-741, Iran; ali.asimi@stu.yazd.ac.ir (A.A.); khgharibi@yazd.ac.ir (K.G.); 2 Bafgh Zinc Smelting Company (BZSC), Yazd 89195-741, Iran; abkhoshkemad@yahoo.com; 3 Chemical Engineering Department, Laval University, Québec, QC G1V 0A6, Canada; moosakazemifarhad@gmail.com; 4 Beneficiation and Hydrometallurgy Research Group, Mineral Processing Research Center, Academic Center for Education, Culture and Research (ACECR) on TMU, Tehran 15119-43943, Iran; 5 Minerals and Metallurgical Engineering, Dept. of Civil, Environmental and Natural Resources Engineering, Luleå University of Technology, SE-971 87 Luleå, Sweden

**Keywords:** jarosite precipitation, zinc calcine, iron removal

## Abstract

Jarosite precipitation process (JPP) is the most frequently used procedure for iron removal in the hydrometallurgical zinc extraction process. However, there is a gap in the knowledge of the relationship between operational parameters and the low contaminant JPP on the industrial scale. This study will address these issues by investigating the behavior of zinc calcine (ZC) as a neutralizing agent, exploring the source of zinc and iron through leaching experiments, and simulating the Jarosite process of the Bafgh Zinc Smelting Company (BZSC). The results showed that the zinc dissolution efficiency was 90.3% at 90 °C, and 73% of the iron present in the calcine can be solubilized. The main outcome was the iron removal of about 85% by alkaline ions present in ZC without the addition of any precipitating agent. The second target was to evaluate the effect of operational parameters on jarosite precipitation. Results revealed that increasing the temperature to 90 °C and the stirring rate to 500 RPM as well as adjusting the ZC’s pH during the jarosite precipitation remarkably improved iron removal. Considering all these factors in the plant could improve Fe precipitation to around 80% on average.

## 1. Introduction

As the second most plentiful element on planet earth (after aluminum), and the fourth most abundant element in the earth’s solid crust (after oxygen, silicon, and aluminum), iron is characteristically accompanying with, not only the crystal structure of over than 600 ores, but also the concentrates of many valuable nonferrous metals such as copper, nickel, zinc, lead, aluminum, manganese, and titanium. Due to the presence of iron as an impurity, iron removal procedures inevitably play a significant role in the hydrometallurgical process of producing these metals [1,2].

Even though, in the hydrometallurgical industries, the hematite, goethite, and paragoethite processes are frequently used as an effective means of eliminating iron from solutions [3,4,5], the Jarosite precipitation process (JPP) is the most well-known and extensively used iron removal method that has remarkable advantages, such as easy operation, low cost, and readily filterability [6,7,8,9,10]. The initial patents of JPP were published by Asturiana de Zinc, Norzinc, and Electrolytic Zinc Company of Australasia [10]. This technology was the first iron removal technique that introduced the commercial production of a readily filterable iron residue in hydrometallurgy and is the most commonly used procedure in different industries such as cement [11], copper [10,12], cobalt [13,14], manganese [15,16], nickel [17,18], and zinc [19,20,21]. During the JPP, Fe^3+^ precipitates gradually from weak acidic sulfate solutions in the form of crystalized AFe_3_(SO_4_)_2_(OH)_6_ under high temperatures. Equation (1) describes a typical reaction for the JPP where A typically stands for potassium, sodium, hydronium, or ammonium. Other ions such as Tl^+^, Pb^2+^, or Ag^+^ can be situated in A-sites as well [22,23,24,25]. Additionally, Fe^3+^ can be replaced by other trivalent cations such as Al^3+^, Ga^3+^, or Cr^3+^ [26,27].

(1) $$3{\mathrm{Fe}}^{3+}+A^{+}+2{\mathrm{SO}}_{4}^{2-}+6H_{2}O\to AFe_{3}{\left(SO_{4}\right)}_{2}{\left(OH\right)}_{6}+6H^{+}$$

In the hydrometallurgical extraction of zinc, JPP has been frequently applied for removing iron from the sulfate liquors before metal recovery through electrowinning. Worldwide, zinc production relies chiefly on the Roasting-Leaching-Electrowinning (RLE) process [28,29]. Zinc production plants mostly utilize the JPP method to remove a high amount of iron, which introduces the zinc sulfate solution in the leaching step. Therefore, several studies since the early 1970s have been conducted about iron control and the JPP in zinc hydrometallurgy. These studies have investigated the history of using the jarosite process [30,31,32,33], its advantages [19,34], and affecting parameters on the process [35,36,37,38]. 

**Figure 1:**
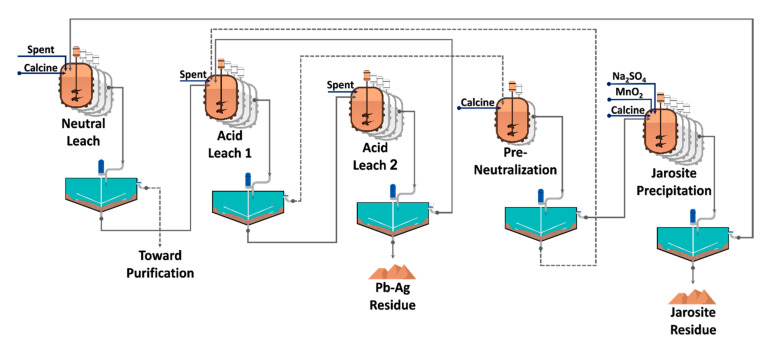
Schematic flow sheet of Bafgh Zinc Smelting Company’s (BZSC) leaching unit and jarosite precipitation line.

Almost all of those investigations used synthetic solutions for their studies. Therefore, there is a considerable gap in JPP industrial conditions, such as the neutralizing agent’s effect on the solution and thermal limitations. This study aims to address these gaps and explore the efficient operational parameters on the JPP for the range of conditions operating at Bafgh Zinc Smelting Company’s (BZSC) production line (Figure 1). Since zinc calcine (ZC), a prevalent neutralizing agent, has many economic and technical advantages for the JPP, its dissolution, and consequent iron precipitation have also been investigated.

## 2. Materials and Methods 

### 2.1. Characterization of Materials and Reagents

BZSC (Yazd, Iran) with 30,000 t/y production has operated the JPP since 2000 [39]. Sulfuric acid and ZC were supplied from the sulfuric acid plant and the roasting unit at BZSC, respectively. The industrial solution for precipitation experiments was also obtained from the input zinc sulfate solution for the plant’s iron removal stage. Industrial grade chemical additives (Na_2_SO_4_ and MnO_2_) were utilized for all experiments. In experiments requiring precipitation seed use, the jarosite cake produced at BZSC was utilized. In this case, after crushing, grinding, and sieving, sodium jarosite residue (<74 µm) was prepared. Chemical compositions of the ZC and zinc sulfate solution are presented in Table 1, respectively. Since ferric ion is the only iron state necessary for the reaction of jarosite precipitation (Equation (1)). Enough MnO_2_ was used to oxidize ferrous ions to ferric ones.

### 2.2. Experimental Procedures

For minimizing the precipitation during the heat-up stage in all experiments, solutions were heated quickly on a hot plate to about the desired reaction temperature. Subsequently, the hot solution was immediately transferred to a baffled 2-L glass reaction vessel in a temperature-controlled oil bath (±1 °C). The glass reactor was equipped with an agitator, a thermometer, and a sampler. Right after the addition of reagents to the solution with a determined temperature, the time of the process was recorded. Two 45° pitched-blade impellers stirred solutions with a 5.5-cm diameter. Samples were periodically withdrawn with syringe filters at predetermined times and quickly cooled to the room temperature to avoid a further reaction. The solution samples of the syringe were transferred to a stoppered test tube to reduce evaporation. After cooling, a 5-mL solution sample was taken and analyzed. At the end of the experiments, the remaining slurry was filtered and washed with warm distilled water and dried in an oven at 110 °C for 24 h. Acid and zinc concentrations of the solutions were measured by NaOH and EDTA (Ethylenediaminetetraacetic acid) titration, respectively. In addition, iron concentration was determined using stannous chloride reduction followed by potassium dichromate titration with a sodium diphenyl sulfonic acid indicator (when [Fe^3+^] > 0.1 g/L) or by Varian SpectrAA 220 Atomic Absorption Spectrometer (when [Fe^3+^] < 0.1 g/L, VARIAN, Victoria, Australia).

#### 2.2.1. Leaching

In general, the neutralizing agents such as slaked lime, limestone, and basic zinc sulfate were employed to adjust acidity in the iron precipitation processes [21,33,40]. However, mostly in the zinc production plants, ZC produced in the plant’s roasting unit is used for that purpose [5,41,42]. In the first step, for leaching experiments, the effect of ZC addition on sulfuric acid neutralization, zinc extraction, and iron leaching-precipitation in the governing conditions at BZSC’s jarosite line was studied. Using sulfuric acid and distilled water, a solution with a concentration of 24.75 g/L (similar to the acidity of the inlet JPP solution at BZSC) was prepared (iron and zinc concentrations were zero). All experiments were run for 300 min at a speed of 600 RPM (revolutions per minute).

#### 2.2.2. Precipitation 

Given the limitations and prevailing conditions at the BZSC, precipitation experiments were performed by the real solution obtained from the plant’s JPP unit. By performing precipitation experiments, the effects of various parameters including temperature, pH, Na_2_SO_4_ concentration, jarosite seed amount, stirring speed, and concentration of precipitating agent on the iron removal were investigated. At the beginning of the experiments, the pH was adjusted to the designated value. Regarding the production of sulfuric acid in the jarosite process (Equation (1)), pH was remodified to the initial value at 15-min intervals using ZC during the reactions. The jarosite precipitation (*η*) was calculated according to Equation (2).

(2) $$\eta_{t}\;=\;\frac{m_{0}+m_{ct}-m_{t}}{m_{0}+m_{ct}}$$

where m_0_ (mg/L) is the initial Fe concentration in a jarosite process solution, m_ct_ (mg/L) is Fe concentration added by neutralizing agent (ZC) until time t, and m_t_ (mg/L) is Fe concentration at time t.

## 3. Results and Discussion

### 3.1. Leaching

For investigating ZC’s behavior in terms of sulfuric acid neutralization, zinc dissolution, iron dissolution, and precipitation in the JPP, it was leached in sulfuric acid solution at different temperatures.

#### 3.1.1. Sulfuric Acid Neutralization

**Figure 2:**
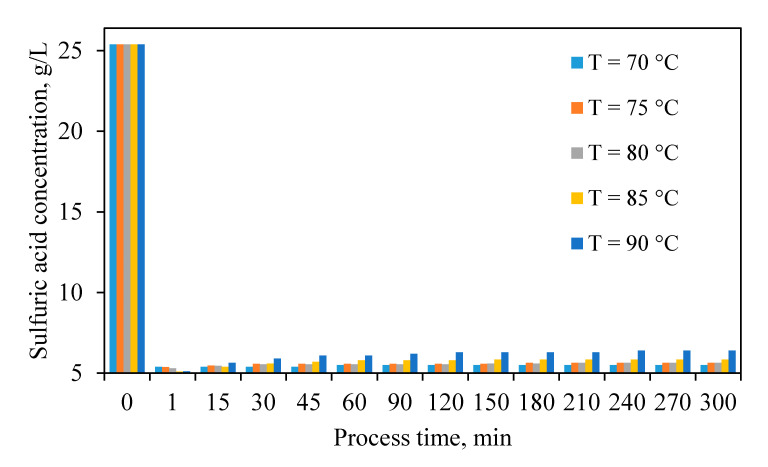
Changes in the concentration of sulfuric acid during leaching experiments.

Results of exploring sulfuric acid neutralization (Figure 2) indicated a rapid decrease in sulfuric acid concentration at the beginning of the experiments. This may have occurred because the majority portion of the metal oxides (mainly zinc and iron oxides) and other alkaline oxides in the neutralizing agent dissolved quite readily in the sulfuric acid based on Equation (3). The ZC dissolution is predominantly dependent on the temperature, and its dissolution increases with a rising temperature. These results also showed that, after a sharp decrease in the sulfuric acid concentration at the initial step of the experiments, sulfuric acid concentration slightly increased. This increase is more visible for higher temperatures and can be due to the formation of jarosite. This is followed by the removal of iron from the solution, which, according to Equation (1), generates H^+^ in the solution (Figure 1).

(3) $$ZnO+H_{2}SO_{4}\to ZnSO_{4}+H_{2}O$$

#### 3.1.2. Zinc Dissolution

**Figure 3:**
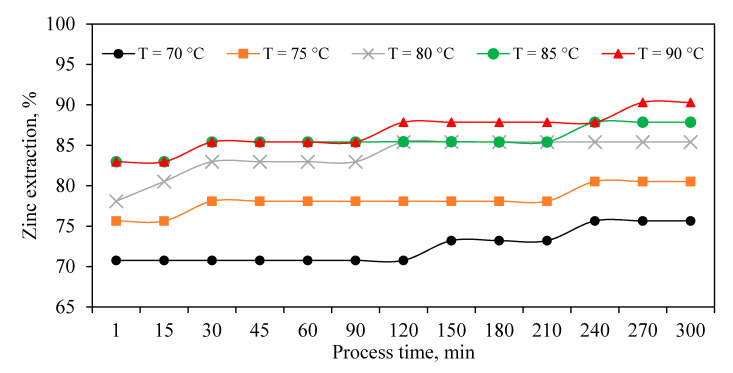
Zinc dissolution efficiency during leaching experiments.

Optimization of zinc dissolution has an important economic point for the plant since the final cake obtained from the JPP in the zinc plants is removed from the processing circuit and stockpiled as a tailing. Exploring zinc extraction from ZC shows (Figure 3) that more than 70% of Zn was extracted after just 1 min since the process has been started and indicated a high rate of dissolution in all temperatures. This phenomenon is in good agreement with the data obtained in experiments evaluating acid concentration (Figure 2). These results (Figure 3) also show the temperature has a significant role in the reaction of Zn dissolution, where the maximum extractions occurred at 85–90 °C.

#### 3.1.3. Iron Dissolution and Precipitation

**Figure 4:**
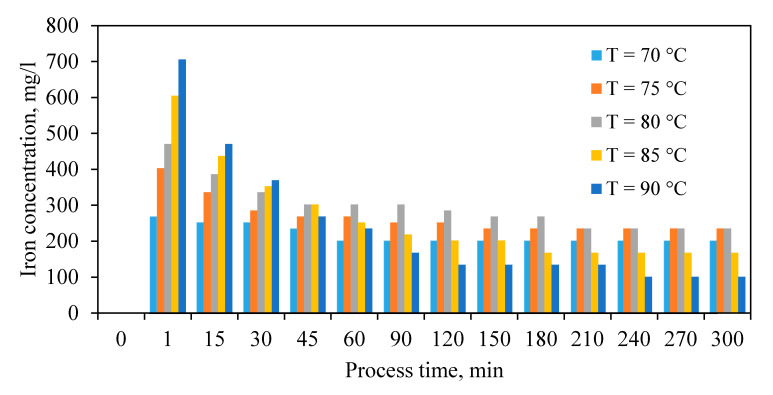
Changes in the concentration of iron during leaching experiments.

Exploring variations of iron concentration as a result of leaching experiments (Figure 4) illustrates that iron leaching increased at the initial stage (<1 min) of the experiment due to the dissolution of the neutralizing agent. Subsequently, iron concentration gradually decreased along with the formation of jarosite and removal of iron from the solution. Even though, by increasing the temperature of the reaction, initially more iron dissolved, and the conditions for the reaction of iron removal were favored. In optimum conditions for ZC leaching, 85% of the dissolved iron was removed from the leaching solution in 240 min. The results of these experiments clearly express that a simultaneous leaching-precipitation process occurs when using only ZC. Therefore, ZC addition modifies the pH for the final jarosite precipitation and acts as another major role by sole precipitation of its own iron content to the tailing.

### 3.2. Precipitation 

Considering the results of preliminary and ZC leaching experiments and, by incorporating the conditions and limitations of the jarosite precipitation line at BZSC, this series of experiments is conducted by changing the parameter values in a reasonable range for the plant. According to the plant operating instructions, the purpose of the JPP is not to remove all the iron present in the solution. In addition, 10–20% of the initial iron concentration must remain in the solution and transfer to the neutral leaching step (Figure 1) to remove some impurities in conjunction with iron in the co-precipitation process. By adding ZC and increasing the solution’s pH to around 5.2, the remaining iron is precipitated in the form of gelatinous Fe(OH)_3_ and co-precipitates some of the impurities such as arsenic, antimony, aluminum, indium, gallium, and germanium [43]. In this section, various parameters, including temperature, pH, precipitating agent concentration, jarosite seed amount, and stirring speed, were examined.

#### 3.2.1. Temperature

**Figure 5:**
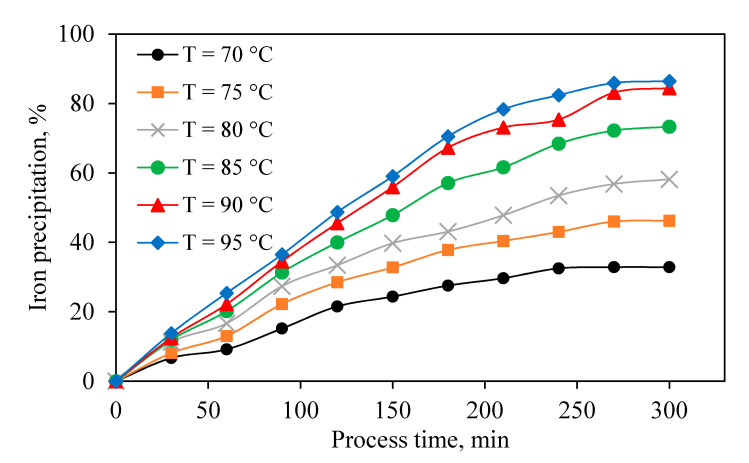
Effect of temperature on iron precipitation (Constant pH = 1, [Na_2_SO_4_] = 2 g/L, stirring speed = 600 RPM).

Temperature is recognized to have a significant effect on both the amount and rate of jarosite precipitation [36]. Exploring the effect of temperature as a key parameter on the jarosite precipitation (Figure 5) shows that, as the temperature increased, the rate of jarosite formation and iron precipitation improved. According to literature, the optimum reaction temperature for jarosite precipitation is 90–100 °C [16,24,44,45]. Due to these results and operational limitations at BZSC, the optimum temperature of 90 °C was chosen for further experiments.

#### 3.2.2. pH

**Figure 6:**
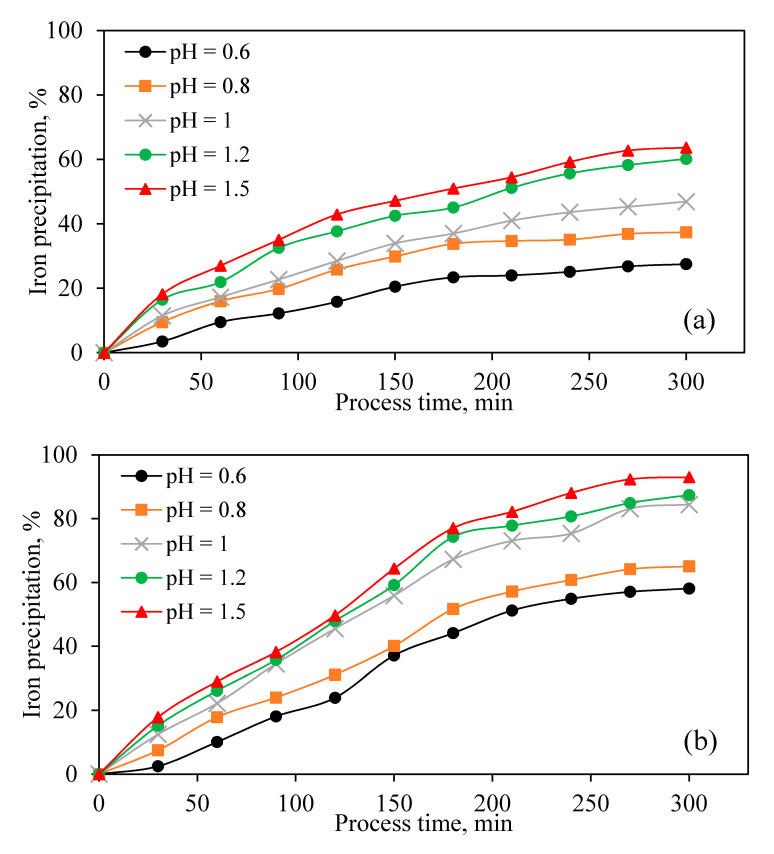
Effect of (**a**) initial pH and (**b**) constant pH on iron precipitation (Temperature = 90 °C, [Na_2_SO_4_] = 2 g/L, Stirring speed = 600 RPM).

pH is one of the most important parameters for the formation of jarosite-type compounds [46,47]. It is necessary to control the remaining acidity from the hot acid leach step and acid, which is produced during the jarosite precipitation reaction (Equation (1)). For this purpose, ZC was used as the neutralizing agent. Based on Equation (1), for each iron mole that precipitated, two moles of hydrogen ions (H^+^) were formed. Thus, there is a necessity to neutralize the released sulfuric acid to enable efficient iron removal. Figure 6a shows the effect of the initial pHs on the JPP. Figure 6b shows the results of iron precipitation in constant pH, which was modified by ZC at certain times. In both sets of experiments, the iron precipitation increased with decreasing sulfuric acid concentration. However, constant modification of pH in the jarosite process is beneficial for iron removal. 

ZnO is the chief compound of ZC that consumes sulfuric acid. However, the ZC also contains some other compounds, which are not dissolved and eventually would be introduced to the jarosite residue. Zinc ferrite (ZnOFe_2_O_3_) is the main compound of ZC containing Zn. It does not dissolve under the jarosite process conditions, and its rejection of the tailings causes zinc loss [48]. Based on BZSC’s process instruction, about 20% of the iron content, which presents in the solution, is required to remain in the solution to remove other impurities in the neutral leach step. Figure 6b shows that the higher the solution’s pH, the more iron removal there is. However, increasing pH results in rejecting more zinc to the tailing. By considering this fact, the JPP should be conducted in the lowest possible pH to reduce zinc losses by decreasing ZC consumption. For this purpose, according to the results (Figure 6b), other precipitation experiments were conducted at a pH of 1.

#### 3.2.3. Na_2_SO_4_ Concentration

**Figure 7:**
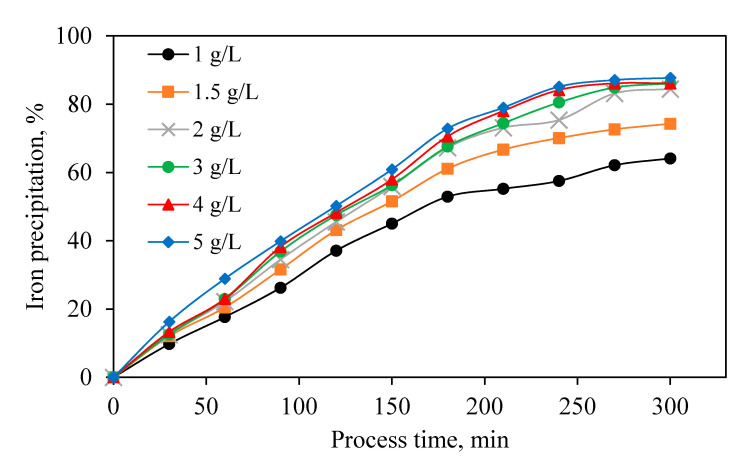
Effect of Na_2_SO_4_ concentration on iron precipitation (Constant pH = 1, Temperature = 90 °C, Stirring speed = 600 RPM).

Ammonium sulfate and sodium sulfate are common additives that provide the alkaline ions required for the JPP [49]. Due to its availability and affordability, sodium sulfate has been used in the production line of the BZSC. JPP was studied in the presence of different amounts of Na_2_SO_4_ as the precipitating agent (Figure 7). It is clear that the rate of the jarosite precipitation is affected by the Na_2_SO_4_ concentration. Since the concentration of Na_2_SO_4_ in the solution increased, the amount of precipitated jarosite increased, and this trend continues with increasing Na_2_SO_4_ concentration to approximately 2 g/L. Thereafter, an additional increase in the concentration of Na_2_SO_4_, has a comparatively minor effect on the amount of iron removal. It is sufficient to remove about 80% of the iron present in the solution as mentioned earlier. For this reason, taking into account the economic aspects of the jarosite process, 2 g/L Na_2_SO_4_ was chosen as the optimum concentration.

#### 3.2.4. Jarosite Seed Amount

**Figure 8:**
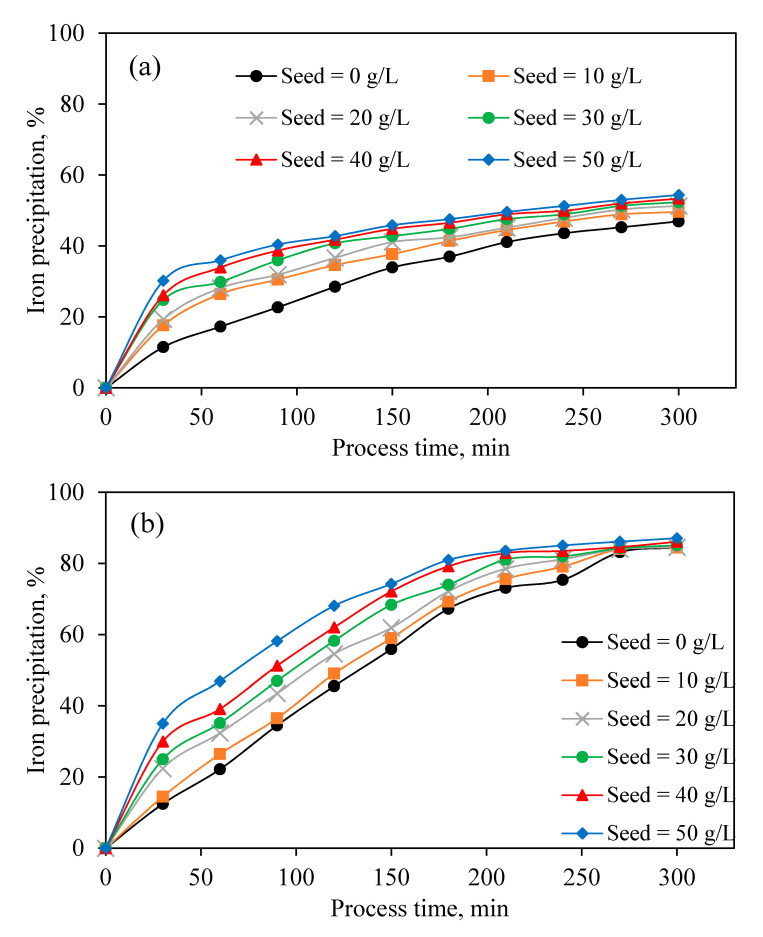
Effect of jarosite seed amount on iron precipitation: (**a**) pH_initial_ = 1, (**b**) Constant pH = 1, (Constant pH = 1, [Na_2_SO_4_] = 2 g/L, Temperature = 90 °C, Stirring speed = 600 RPM).

It was reported that the initial presence of jarosite seed could effectively accelerate the iron precipitation, and it does not alter the jarosite reaction equilibrium [49]. The results of the precipitation experiments in the presence of different amounts of jarosite seed at 90 °C (Figure 8a) illustrates the effect of various jarosite seed amounts on iron precipitation efficiency when the initial pH is set to 1, while the results in Figure 8b were obtained in constant pH of 1. It is observed that, in both sets of experiments, increasing the initial amount of jarosite seed promotes the iron precipitation rate. The results of experiments conducted at an initial pH of 1 indicate an increase in the rate of iron precipitation where the precipitation generally increased. However, in constant pH, the rate of precipitation differs among all seed amounts, and total precipitation is independent of the amount of jarosite seed. Keeping the amount jarosite seed as low as possible leads to a lower volume of tailing. Thus, a lower dissolved Zn would be lost. 

#### 3.2.5. Stirring Speed

**Figure 9:**
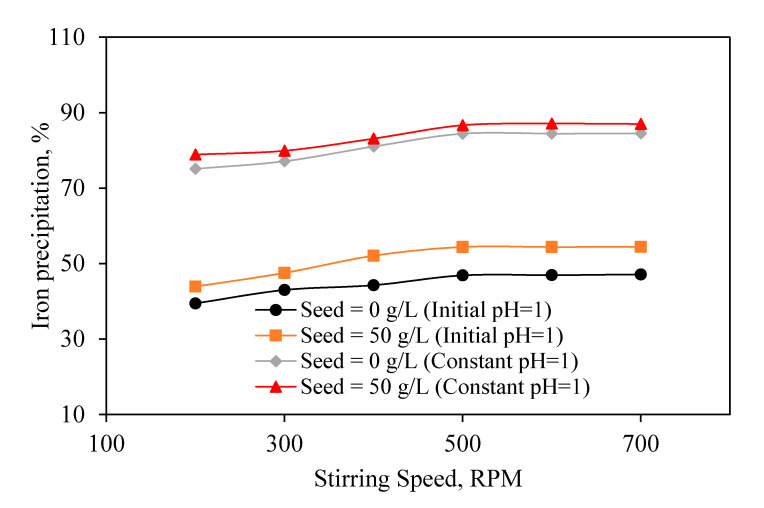
Effect of stirring speed on iron precipitation (Process time of 300 min). (Constant pH = 1, [Na_2_SO_4_] = 2 g/L, Temperature = 90 °C).

Examining the effect of stirring speed on iron precipitation in both constant pH and varied pH experiments after 300 min (Figure 9) shows, in all experiments, the addition of jarosite seed had a positive effect on total precipitation. In addition, any increase in stirring speed until 500 RPM leads to an improvement in the iron precipitation.

**Figure 10:**
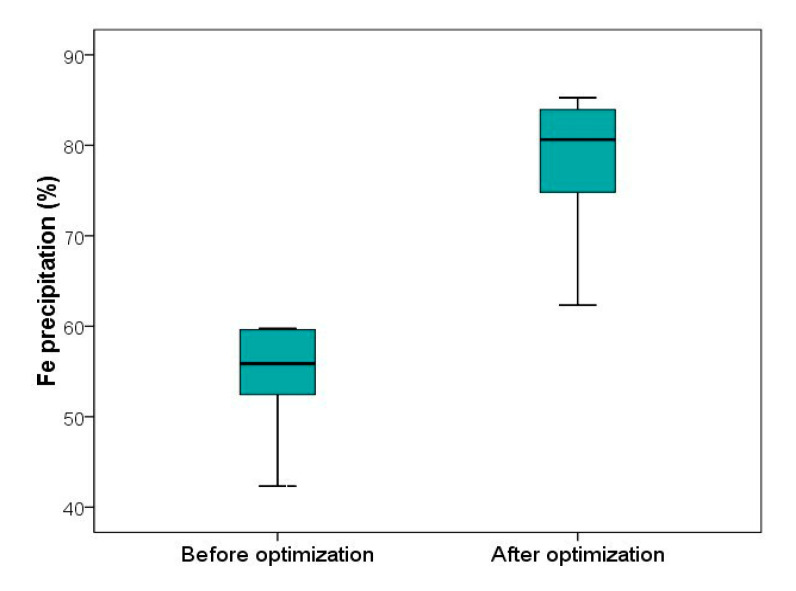
A comparison between Fe precipitation before and after optimization in the Jarosite precipitation process (JPP) of the BZSC plant.

Considering all these optimum conditions in the BZSC plant and comparing the results (Figure 10) of a couple of months of monitoring before and after these implementations indicated that the optimization process was very successful and could improve Fe precipitation to around 80% on average.

## 4. Conclusions

The effect of various parameters on zinc dissolution and JPP was investigated based on operational conditions in an industrial production line. In the first step, within leaching experiments, ZC as the most common and suitable neutralizing agent was solely used to evaluate its own effect on the acid neutralization, zinc dissolution, iron dissolution, and possible iron precipitation. Results of calcine leaching experiments indicated that most of the neutralizing agents dissolved right away in the sulfuric acid, causing the release of various elements into the solution, especially zinc and iron, and consuming sulfuric acid. Immediately after iron dissolution, precipitation of this element occurred due to an increase in pH and the possible presence of alkaline elements originating from ZC. Increasing reaction temperature from 70 to 90 °C increased and accelerated the zinc and iron extraction rate. In the optimum temperature (90 °C), 87% of zinc in the calcine was extracted, 85% of dissolved iron was precipitated, and 76% of sulfuric acid was neutralized. In the second stage of this study, the effect of efficient variables including temperature, pH, Na_2_SO_4_ addition, jarosite seed, and stirring speed on iron removal by JPP was investigated for industrial solution obtained from BZSC. The most influential parameters were temperature, pH, and stirring speed, among others. Increasing temperature from 70 to 90 °C significantly improved the iron precipitation. It was observed that higher pH of the solution results in higher iron removal. However, zinc dissolution mainly from zinc ferrite would be suppressed and consequently rejected to the tailing. Thus, there should be a tradeoff between the amount of iron removed and zinc loss. In this study, pH of 1 was suggested. Moreover, higher iron precipitation was observed when pH was constantly modified to the initial value compared with the cases without pH recodification. This is due to the fact that alkaline matters were always available to continue the precipitation process. The addition of Na_2_SO_4_ as a neutralization agent also had a positive effect on iron removal. An increasing amount of this agent to 2 g/L improved iron removal, and its further addition had no beneficial effect. The jarosite application as a seeding agent only improved the rate of iron removal and did not impact the total iron precipitation. It was concluded that iron removal depends on stirring speed, and increasing this variable to 500 RPM enhanced the process. Implementing all these factors could improve Fe precipitation to around 80% in the plant.

## Figures and Tables

**Table 1:** Chemical composition of the zinc calcine and zinc sulfate solution.

**Zinc Calcine (wt.%)**											
Zn	Fe		Pb		Ca		K		Na		Ag
51.3	3.70		3.21		1.89		0.22		0.26		0.01
**Zinc Sulfate Solution (g/L)**											
Zn		Fe (Total)		Fe^2+^		K		Na		H_2_SO_4_	
101.37		9.173		0.094		0.173		0.396		24.75	
